# Multisensory System for the Detection and Localization of Peripheral Subcutaneous Veins

**DOI:** 10.3390/s17040897

**Published:** 2017-04-19

**Authors:** Roemi Fernández, Manuel Armada

**Affiliations:** Centre for Automation and Robotics (CAR) CSIC-UPM, Carretera de Campo Real, Km. 0,200. La Poveda, Arganda del Rey, 28500 Madrid, Spain; manuel.armada@csic.es

**Keywords:** SWIR camera, TOF camera, peripheral vein detection, subcutaneous vein localization, automatic catheter insertion

## Abstract

This paper proposes a multisensory system for the detection and localization of peripheral subcutaneous veins, as a first step for achieving automatic robotic insertion of catheters in the near future. The multisensory system is based on the combination of a SWIR (Short-Wave Infrared) camera, a TOF (Time-Of-Flight) camera and a NIR (Near Infrared) lighting source. The associated algorithm consists of two main parts: one devoted to the features extraction from the SWIR image, and another envisaged for the registration of the range data provided by the TOF camera, with the SWIR image and the results of the peripheral veins detection. In this way, the detected subcutaneous veins are mapped onto the 3D reconstructed surface, providing a full representation of the region of interest for the automatic catheter insertion. Several experimental tests were carried out in order to evaluate the capabilities of the presented approach. Preliminary results demonstrate the feasibility of the proposed design and highlight the potential benefits of the solution.

## 1. Introduction

It is estimated that about 90–95% of patients in hospitals receive some kind of intravenous therapy. In comparison with other methods of administration, the intravenous technique is the fastest means of providing fluids and medications throughout the human body. That is the reason why it is widely used to transfuse blood and blood products, provide parenteral nutrition, correct dehydration and electrolyte imbalances, deliver medication and chemotherapeutic agents, and provide avenues for dialysis/apheresis, hemodynamic monitoring, and diagnostic testing [1].

Within intravenous therapy, the peripheral venous catheter is the most frequently used vascular access device. It is applied to most emergency room and surgical patients, and before some radiological imaging techniques using radiocontrast, resulting in more than a billion peripheral intravenous catheters being used per year worldwide [2]. The procedure implies inserting a catheter into the vein by a needle, which is subsequently removed while the small tube of the cannula remains in. The process involves then a very demanding motor coordination, requiring proper training and significant experience, especially when dealing with children and the elderly. Even so, errors are very common, and often, nursing staff has to try several times before to place the needle successfully, which causes pain and distress to the patient, and frustration to the clinicians. Replacement of catheters and rotation of site is recommended every 72 to 96 h in order to reduce the risk of phlebitis and bloodstream infection [3,4,5], which means repeating the process several times in case of prolonged stays in the hospital. In addition, the insertion of peripheral intravenous catheters exposes health care workers to certain occupational risks, such as a needlestick injury and exposure to blood, which can lead to bloodborne infections [6,7].

In the last decade, several robotic systems have been integrated successfully in the medical field, offering objective and measurable advantages in comparison with traditional procedures, mainly due to the high accuracy and repeatability of their actions [8,9]. These features also make robotic systems stand out as an alternative to provide faster, safer and less painful insertion of intravenous catheters [10,11,12]. One fundamental step to attain such systems is the effective detection and localization of target subcutaneous veins.

The current state of the art related to automatic detection of veins is predominantly focused on the recognition of finger-vein patterns that can be used as biometric signatures in personal identity authentication systems [13]. For instance, in [14], the repeated tracking of dark lines on an image of a finger captured under infrared light is proposed for finger-vein pattern extraction. In [15], the same authors present an improved method based on the calculation of local maximum curvatures in cross-sectional profiles of the image. In [16], a mean curvature method, which uses geometrical properties of the intensity field to find alley-like structures with negative mean curvatures is proposed for the same purpose. In [17], authors present a finger vein extraction method using gradient normalization and principal curvature calculation. In [18], a finger-vein pattern identification system based on PCA for image pre-processing and feature extraction using LDA is described. Finger-vein image enhancement is proposed in [19] by using a fuzzy-based fusion method with Gabor and Retinex filtering, whereas in [20], authors propose a new finger-vein capturing device that ensures accurate finger positioning to reduce misalignment when veins images are captured. All these methods have in common the fact that they are based on an image of a finger illuminated with an infrared light and acquired by a CCD camera.

Vein identification for automated intravenous drug delivery was proposed in [21]. The system consisted of a web-camera, a near-infrared light and a Digital Single Lens Reflex (DSLR) camera with an external filter to block visible light. The authors concluded that better results are achieved for the images captured with the DSLR and poor results for images obtained with the web-camera can be attributed to the low contrast quality of these images.

Multispectral imaging systems and NIR spectroscopy have also been considered by several authors. Subcutaneous vein detection using multispectral imaging is proposed in [22]. In this case, visible and NIR images acquired by a multispectral imager are used in a normalized subtraction algorithm for improving contrast, and consequently the vein detection performance. In [23], near-infrared spectroscopy is proposed for an education-focused mobile medical application devised to help to improve the decision-making skills of healthcare students in venipuncture. There are also three commercial devices approved by the FDA that use NIR spectroscopy to facilitate peripheral intravenous catheter insertion: the VeinViewer [24] (Christie Medical Holdings, Memphis, TN, USA), the AccuVein [25] (AccuVein LLC, Cold Spring Harbor, New York, NY, USA) and the VascuLuminator [26] (De Koningh Medical Systems, Arnhem, The Netherlands). All these solutions are conceived to improve visualization of subcutaneous veins instead of detecting them automatically.

There is scarce literature related to 3D localization of subcutaneous veins. In [11,12,27], authors propose active optical triangulation for range data acquisition and parametric surface modelling to store the 3D shape of the patient arm. Active optical triangulation is achieved by combining a camera and a laser stripe line generator. Worthy of mention is also the research presented in [28]. The proposed system provides augmented vein structures that are back-projected and superimposed on the skin surface of the hand for assisting doctors in locating the injection veins. The system consists of two industrial cameras, a color micro projector, NIR light sources, a support structure with multi-degree of freedom, and an underpan. Veins are segmented by a multiple-feature clustering method. Vein structures captured by the two cameras are matched and reconstructed based on epipolar constraint and homographic property. The skin surface is reconstructed by active structured light with special encoding values. Results show that the system effectively provides augmented display and visualization of subcutaneous veins.

This paper presents an automatic system that combines a SWIR (Short-Wave Infrared) camera, a TOF (Time-Of-Flight) camera, a NIR (Near Infrared) lighting system and the associated algorithm for the detection and localization of peripheral subcutaneous veins. The solution is intended to be used for the future automation of peripheral intravenous catheter insertion.

The rest of the paper is organized as follows: Section 2 describes the design and implementation of the proposed multisensory system and the associated algorithm. Section 3 presents the results obtained from the different experimental tests that have been carried out. Section 4 discusses the main results of this work and finally, Section 5 summarizes the major conclusions.

## 2. Materials and Methods

This section describes the multisensory system that has been designed for the data acquisition and explains the processing algorithm that has been implemented for the automatic detection and localization of peripheral subcutaneous veins.

### 2.1. Multisensory System Description

The proposed multisensory system is based on the combination of a GoldEye P-032 SWIR camera (Allied Vision, Stradtroda, Germany), a SwissRanger SR-400011 TOF 3D camera (Mesa Imaging, Zürich, Switzerland), and a NIR light source that consists of 96 LEDs of 940 nm distributed in two linear arrays. The GoldEye P-032 SWIR camera has a high spectral response from 900 to 1700 nm thanks to its indium gallium arsenide (InGaAs) sensor and features a maximum frame rate of 30 fps at its full resolution of 636 × 508 pixels with 14-bit A/D conversion. The TOF camera provides a depth map and amplitude image at the resolution of 176 × 144 pixels with 16 bit floating-point precision, as well as *x*, *y* and *z* coordinates to each pixel in the depth map. IR light can penetrate human tissues to about 3 to 5 mm subcutaneous depth before losing coherence and directionality to diffusion due to the low optical absorption of human skin and muscles in the NIR region of the electromagnetic spectrum [29,30]. On the contrary, blood is a strong absorber of NIR radiation, increasing the contrast between the subcutaneous veins and the surrounding tissues in NIR images [31,32]. Thus, the SWIR camera enables the acquisition of the required data for the detection of areas of interest that could belong to peripheral subcutaneous veins, whereas the TOF camera supplies simultaneously fast acquisition of accurate distances and intensity images of targets, enabling their localization in the coordinate space. Intrinsic and extrinsic calibration parameters of both cameras were estimated by using the Matlab camera calibrator app (http://www.mathworks.com/ products/matlab/). A distance measurement calibration was also carried out in Matlab for the TOF camera by following the method proposed in [33].

**Figure 1:**
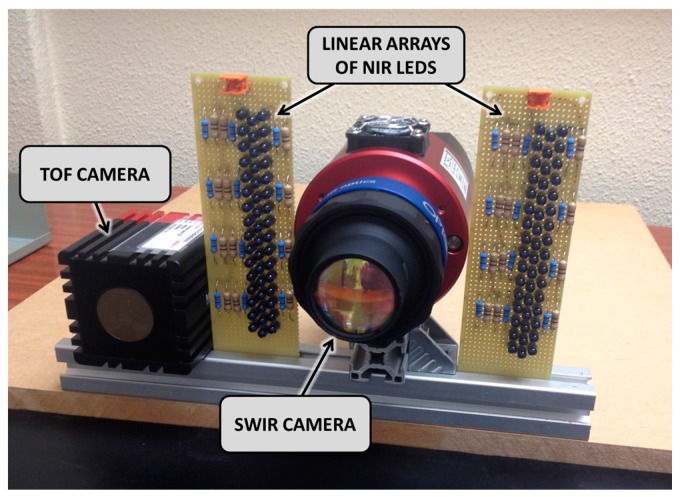
Close-up view of the proposed multisensory system.

Figure 1 shows the layout of the different elements that make up the proposed system. Note that the linear arrays of lEDs are placed side by side with the SWIR camera, so the SWIR camera captures the light reflected by the patient arm.

In addition, the custom-made multisensory rig that integrates the SWIR camera, the TOF camera and the two linear arrays of NIR LEDs is mounted in a Bosch frame, in such a way that the image planes of the cameras are parallel to the table where subjects place their hands or arms for the automatic detection and localization of peripheral subcutaneous veins.

### 2.2. Algorithm for Automatic Detection and Localization of Peripheral Subcutaneous Veins

**Figure 2:**
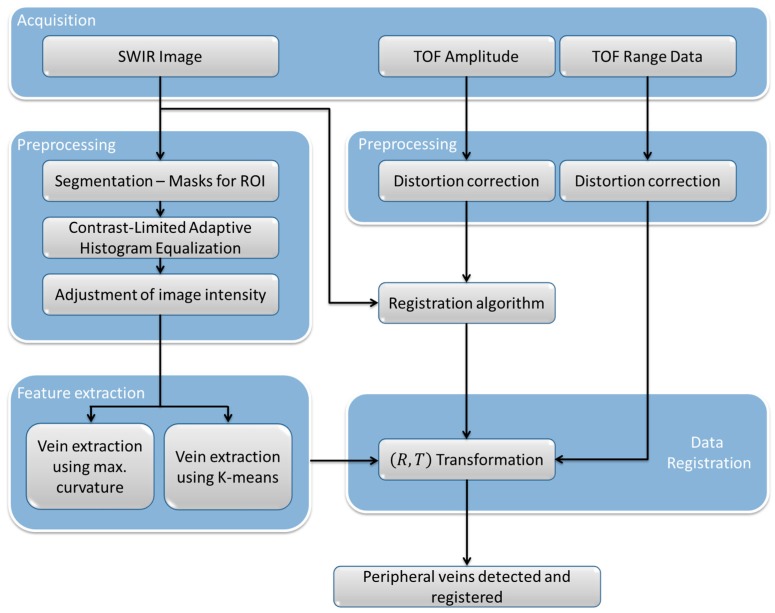
Algorithm for automatic detection and localization of peripheral subcutaneous veins.

Figure 2 shows a block diagram of the proposed algorithm, which consists of two main parts: one devoted to the features extraction from the SWIR image, and another envisaged for the registration of the range data provided by the TOF camera, with the SWIR image and the results of the peripheral veins detection.

For features extraction, the first step involves the segmentation of the acquired SWIR image into two regions, the background and the foreground. The foreground represents the part of the patient’s body where the detection of the peripheral veins is going to be carried out, whereas the background represents the rest of the image pixels that are not required for further processing. Otsu’s method [34] is then utilized for choosing a global threshold that minimizes the intraclass variance of the background and the foreground pixels. With the attained threshold, a binarization of the original SWIR image is conducted, followed by a dilatation of the background region. The aim of this dilatation is discarding those pixels where the transition from background to foreground region takes place, and which can produce detection errors in the subsequent steps. In fact, the dilatation process is performed twice by using two square structuring elements with two different width values, 10 and 15 pixels, respectively. Thus, two masks are obtained and applied to the original SWIR images. In this way, once the vein extraction is accomplished, a logical AND will be applied to both images, eliminating false detections due to the effects produced by the edges during the segmentation process. Thus, the obtained masks ensure that only the region of interest of the original SWIR image is considered for features extraction, increasing the algorithm performance.

Next, contrast-limited adaptive histogram equalization [35] is applied to the masked images for compensating non-uniform lighting conditions, followed by an adjustment of the image intensity values. This last adjustment consists on mapping the intensity values to new values such that 1% of data is saturated at low and high intensities of the SWIR image. In this way, the contrast between the veins and the surrounding tissues on the SWIR image is enhanced. After these preprocessing steps, features extraction is accomplished by two different techniques: the maximum curvature method and the k-means clustering.

The maximum curvature method presented in [15], which is one of the better finger-vein extraction methods [13], is based on the fact that a vein appears like a dent with high curvature in the cross-sectional profile, and consequently, calculates the local maxima of each cross-sectional profile (in four directions: horizontal, vertical and two diagonal directions).

On the other hand, the k-means clustering used in this application partitions the image pixels into three clusters: background, surrounding tissues and subcutaneous veins. From these three clusters, only the subcutaneous veins cluster is considered as final solution. In this case, the centroids of clusters used to characterize the data are determined by minimizing the sum of squad errors given by:

(1) $$J_{K}=\overset{K}{\underset{k=1}{\sum }}\underset{i\in C_{k}}{\sum }{\left(x_{i}-m_{k}\right)}^{2}$$

where $\left(x_{1},\ldots ,x_{n}\right)=X$ is the data matrix, $m_{k}=\sum_{i\in C_{k}}\left(x_{i}/n_{k}\right)$ is the centroid of the cluster $c_{k}$ and $n_{k}$ is the number of points in $C_{k}$ [36].

Once the peripheral subcutaneous veins have been detected on the SWIR image, second part of the algorithm addresses the registration of the detection results with the range data provided by the TOF camera, so that they share a common reference frame. Before registration, radial distortion of the TOF data is corrected. The relationship between the distortion coordinate system and the imaging coordinate system is given by:

(2) $$x_{d}=x\left(1+k_{1}r^{2}+k_{2}r^{4}+k_{3}r^{6}\right)\;y_{d}=y\left(1+k_{1}r^{2}+k_{2}r^{4}+k_{3}r^{6}\right)$$

where $\left(x_{d},y_{d}\right)$ is the distorted coordinate, (x,y) is the normalized imaging coordinate, $k_{1},\;k_{2},\;k_{3}$ are the radial distortion coefficients of the lens, and $r^{2}=x^{2}+y^{2}.$ Normalized image coordinates are calculated from pixel coordinates by translating to the optical center and dividing by the focal length in pixels. Thus, x and y are dimensionless. Radial distortion coefficients are determined with the intrinsic and extrinsic parameters of the camera during the calibration process. This calibration process is carried out once, by taking pictures of different orientations of a planar checkboard considered as a metric reference for the system. The position of all the square corners in a set of 50 pictures was analyzed in order to obtain the camera parameters.

Then, as SWIR image and TOF data are acquired with cameras that exhibit a different pixel array and a different field of view, the random sample consensus (RANSAC) algorithm [37] is adopted for registering the acquired data, in such a way that a direct correspondence between the pixels of the different images is obtained. As relative positions of SWIR and TOF camera remain fixed on the designed set-up, the RANSAC algorithm is used only once and offline, for finding the rotation and the translation (R,T) that enable the mapping of the TOF data into the reference frame of the processed SWIR image, being R a 2×2 matrix and T a 2×1 vector. For that, N pairs of control points’ correspondences between frames $F_{1}$ and $F_{2}$ are selected, where $F_{1}$ and $F_{2}$ correspond to TOF and SWIR frames respectively. In this particular case, five pairs of control points were selected manually in 20 different scenes, resulting in a total of 100 pairs of control points (N=100). The control points are represented by 2D coordinates $\left(X_{1}^{i},X_{2}^{i}\right)$ in their respective reference systems. RANSAC samples the solution space of (R,T) and estimates its fitness by counting the number of inliers, $f_{0}$:

(3) $$f_{0}\left(F_{1},F_{2},R,T\right)=\overset{N}{\underset{i}{\sum }}L\left(X_{1}^{i},X_{2}^{i},R,T\right)$$

where:

(4) $$L\left(X_{1}^{i},X_{2}^{i},R,T\right)=\{\begin{array}{cc} 1, & e=\|RX_{1}^{i}+T-X_{2}^{i}\|<\epsilon \\ 0, & otherwise \end{array}$$

and ϵ is the threshold beneath which a features match $\left(X_{1}^{i},X_{2}^{i}\right)$ is determined to be an inlier. RANSAC chooses the transform with the largest number of inlier matches [38,39]. The resulting (R,T) is then utilized online for matching the range data provided by the TOF camera with the processed SWIR data that contains the resulting peripheral veins detection.

## 3. Results

**Figure 3:**
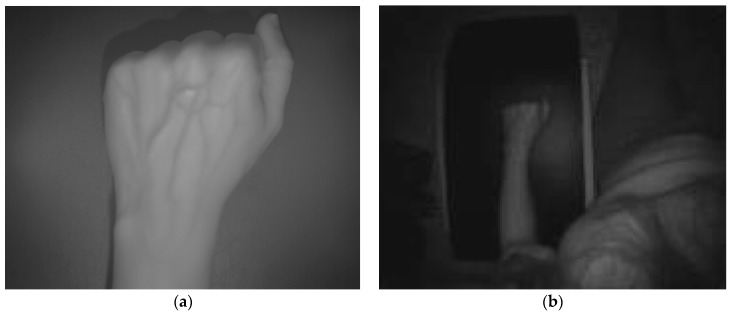
Test 1—Acquired images. (**a**) SWIR image; (**b**) TOF amplitude image.

**Figure 4:**
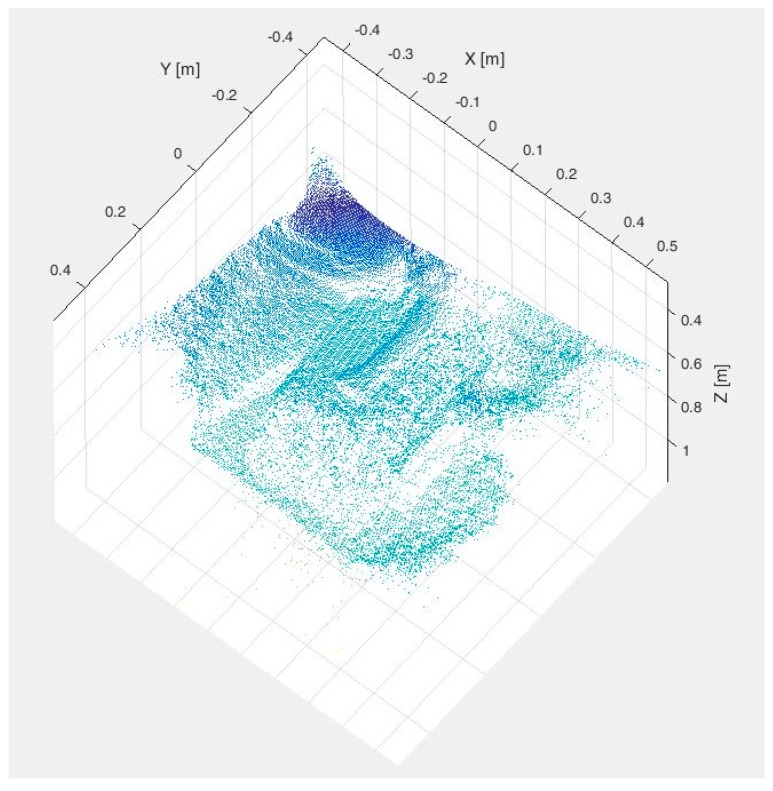
Test 1—Raw point cloud provided by the TOF camera.

In order to validate the proposed multisensory system and the associated algorithm for the detection and localization of peripheral veins, several experimental tests have been carried out. Figure 3 and Figure 4 show the data acquired by the multisensory system for one of the experimental tests performed to evaluate the detection of the peripheral subcutaneous veins on the front of the hand.

Figure 3 presents the SWIR image and the TOF amplitude image, whereas Figure 4 displays the raw point cloud provided by the TOF camera.

**Figure 5:**
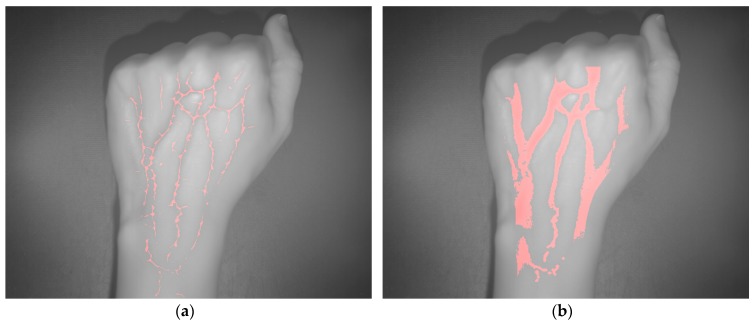
Test 1—Vein extraction. (**a**) Results from applying the proposed preprocessing and the adapted maximum curvature method; (**b**) Results from applying the method based on the k-means clustering.

The vein extraction results achieved after applying the proposed algorithms to the acquired SWIR image are presented on Figure 5. Left-hand side image displays the result from the adapted maximum curvature method, while right-hand side image shows result obtained with the method based on the k-means clustering. Red color is utilized to visualize pixels identified or classified as vein.

**Figure 6:**
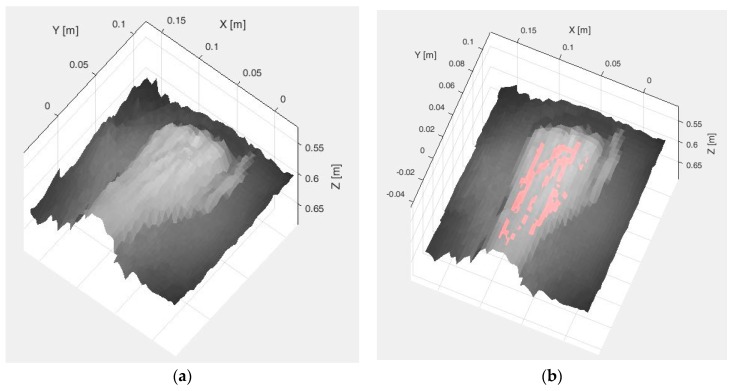
Test 1. (**a**) SWIR-D visualization; (**b**) Registered SWIR image with vein detection results.

Once the vein extraction is completed, TOF data are registered in order to locate the veins spatially. Figure 6a shows the result from registering the original SWIR image with the TOF data (termed SWIR-D visualization, as it combines the SWIR information with the estimated depth for each pixel), whereas Figure 6b displays the 3D mapping of the veins detected with the method based on the k-means clustering after applying the proposed registration algorithm.

**Figure 7:**
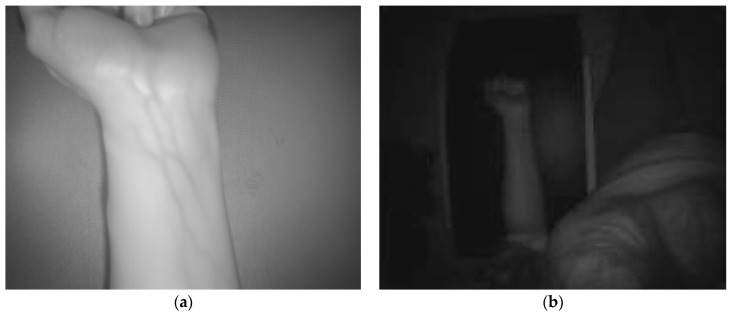
Test 2 - Acquired images. (**a**) SWIR image; (**b**) TOF amplitude image.

**Figure 8:**
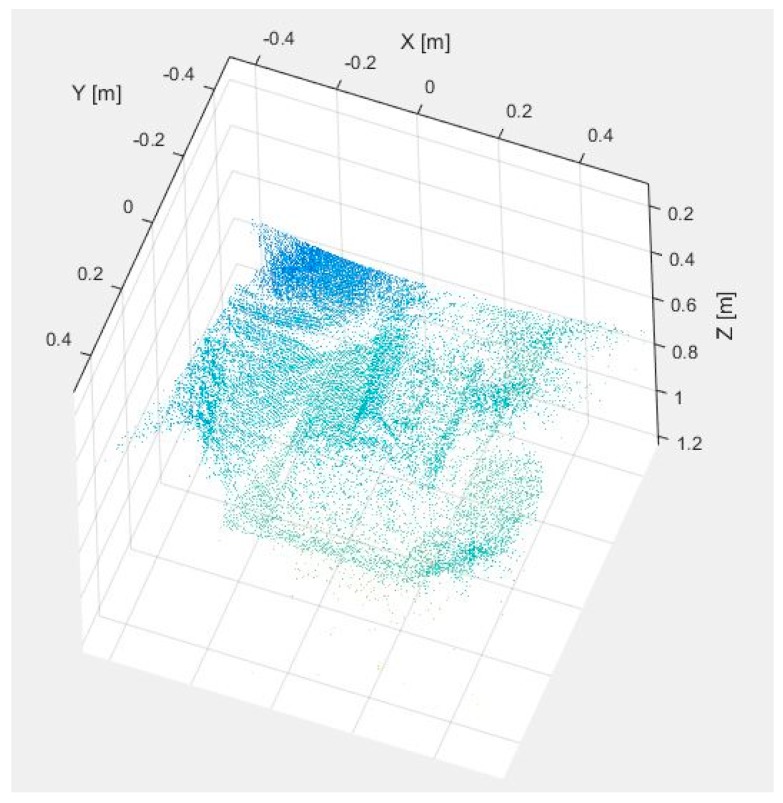
Test 2—Raw point cloud provided by the TOF camera.

Figure 7 and Figure 8 display the dataset acquired with the proposed multisensory system for a second test scene. In this case, it is desired to evaluate the detection of the subcutaneous veins on the wrist anterior view. The dataset includes the SWIR image (Figure 7a), the TOF amplitude image (Figure 7b) and the raw point cloud provided by the TOF camera (Figure 8).

**Figure 9:**
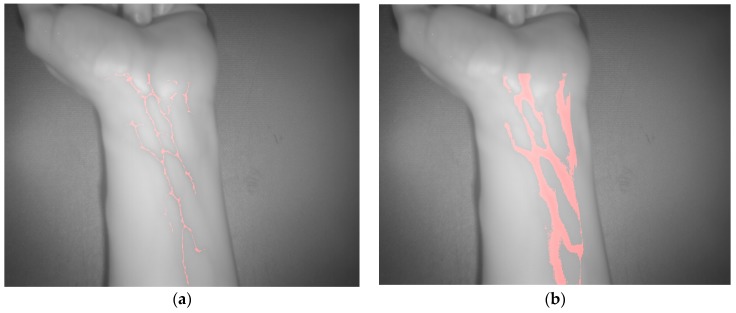
Test 2—Vein extraction. (**a**) Results from applying the proposed preprocessing and the adapted maximum curvature method; (**b**) Results from applying the method based on the k-means clustering.

**Figure 10:**
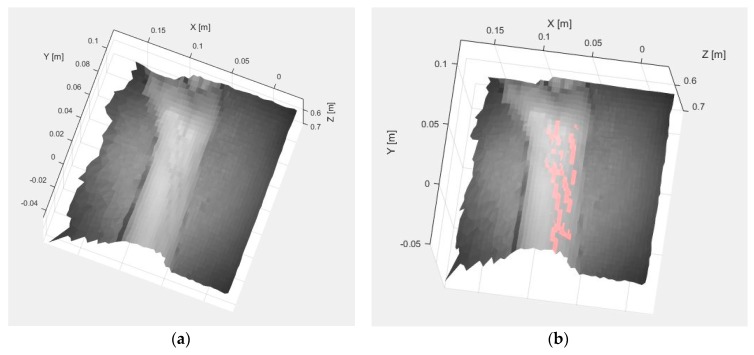
Test 2. (**a**) SWIR-D visualization; (**b**) Registered SWIR image with vein detection results.

**Figure 11:**
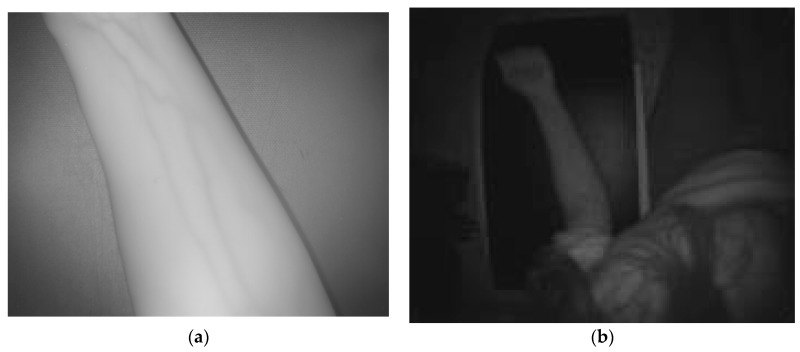
Test 3—Acquired images. (**a**) SWIR image; (**b**) TOF amplitude image.

**Figure 12:**
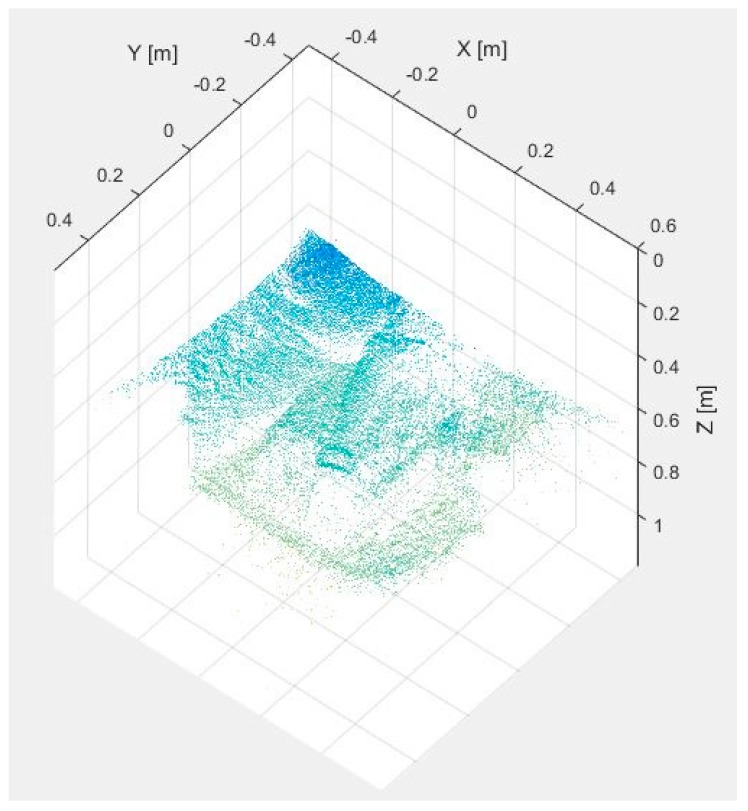
Test 3—Raw point cloud provided by the TOF camera.

Figure 9a,b shows the results obtained after applying the adapted maximum curvature method and the method based on the *k*-mean clustering, respectively. Next, Figure 10a displays the obtained result after registering the TOF data with the original SWIR image, and Figure 10b illustrates the SWIR-D visualization obtained after applying the proposed registration algorithm. In this SWIR-D visualization, the subcutaneous veins detected on the SWIR image with the method based on the k-means clustering are mapped onto the 3D reconstructed surface. Lastly, the third experiment is intended to evaluate the detection and localization of subcutaneous veins on the anterior view of the forearm. Figure 11 and Figure 12 display the dataset acquired with the proposed multisensory system, including a SWIR image (Figure 11a), a TOF amplitude image (Figure 11b) and a raw point cloud provided by the TOF camera (Figure 12).

**Figure 13:**
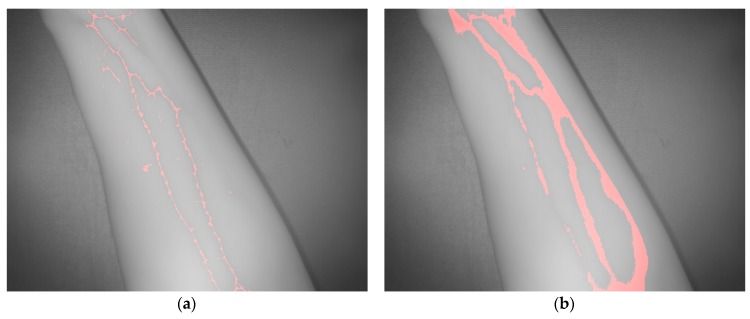
Test 3—Vein extraction. (**a**) Results from applying the proposed preprocessing and the adapted maximum curvature method; (**b**) Results from applying the method based on the k-means clustering.

**Figure 14:**
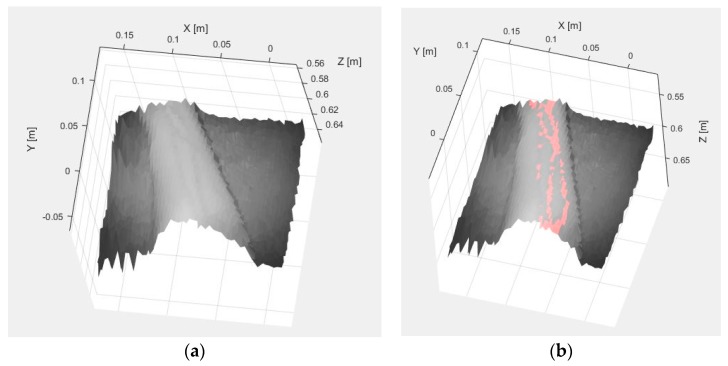
Test 3. (**a**) SWIR-D visualization; (**b**) Registered SWIR image with vein detection results.

Figure 13a,b shows the vein detection results obtained with the adapted maximum curvature method and the method based on the k-means clustering, respectively. Finally, Figure 14a displays the obtained result after registering the TOF data with the original SWIR image, while Figure 14b illustrates the mapping of the veins detected with the method based on the k-means clustering onto the 3D reconstructed surface, after applying the proposed registration algorithm. Note that in the three presented experiments, only the registration results obtained with the detection based on the k-means clustering are displayed. This is due to the fact that the method based on the adapted maximum curvature provides a low number of image pixels identified as vein, which demerits the registration results. On the contrary, the detection method based on the k-means clustering provides a higher a proportion of image pixels that are correctly identified as vein, and consequently, fits better as input for the subsequent registration procedure, improving performance results.

In order to evaluate quantitatively the performance of the proposed approach, ground truth data was carefully collected and produced for the three test scenes presented previously. This process included the manual labelling of the pixels that visually appear to belong to the veins on the SWIR images and the manual measurement of the Cartesian coordinates of ten control points located on the test scenarios. These coordinates were measured with respect to the reference frame located at the intersection of the optical axis with the front face of the TOF camera.

**Figure 15:**
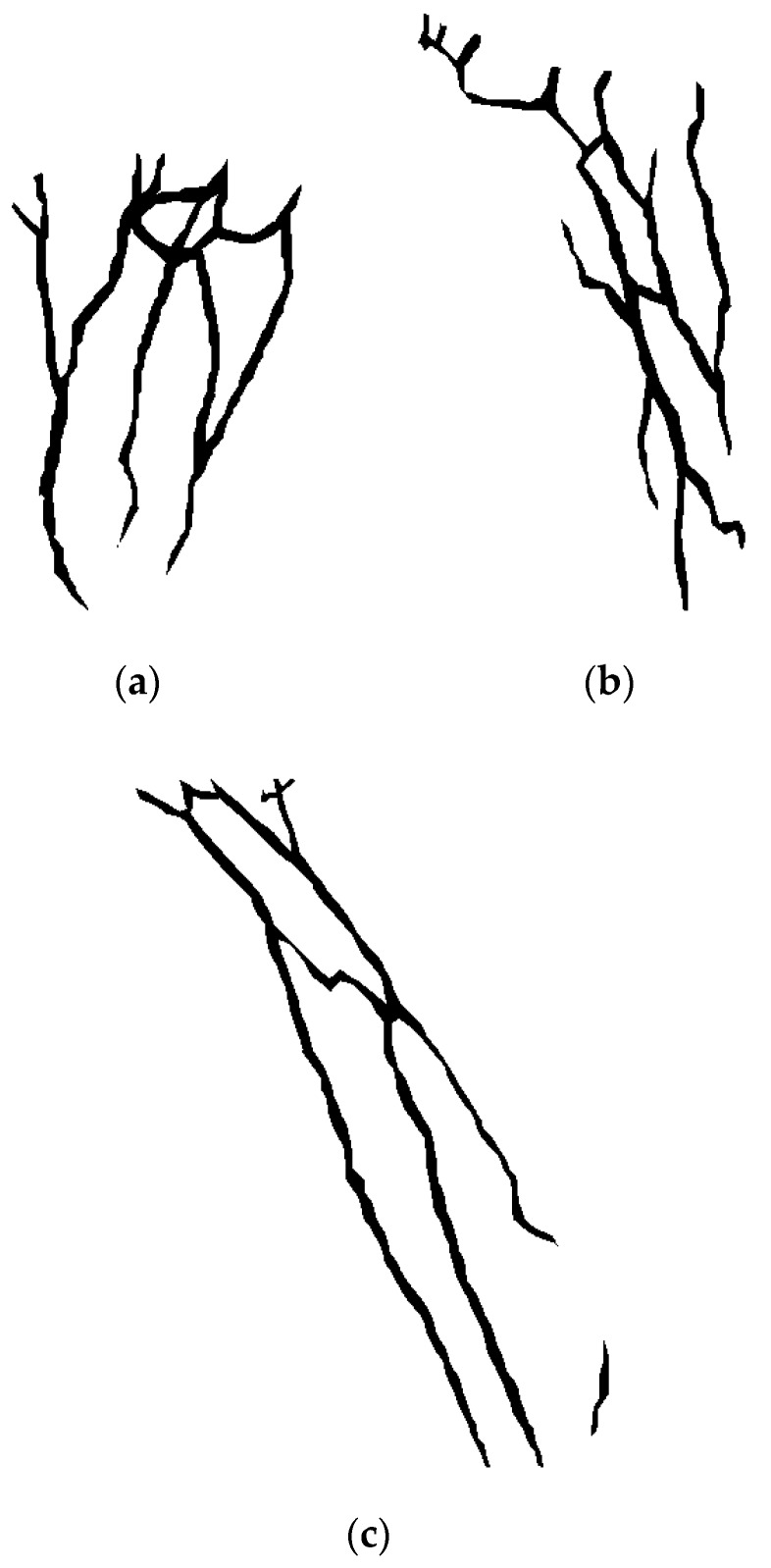
Labelled images. (**a**) Ground truth data for test 1; (**b**) Ground truth data for test 2; (**c**) Ground truth data for test 3.

Figure 15 shows the labelled images for the SWIR images presented on Figure 3a, Figure 7a and Figure 11a. Note that the same masks applied during the segmentation process were utilized after the manual labelling of the SWIR images, in order to get the same regions of interest used during the features extraction. These images were then utilized as ground truth data in the pixel-level comparison carried out with the detection results obtained with the proposed methods. Detection performance is then evaluated in terms of true positive rate, accuracy and total error rate. The true positive vein detection rate, which is a proportion of the pixels that are correctly identified as vein, is defined as:

(5) $$\mathrm{TP}=\frac{\mathrm{number}\;\mathrm{of}\;\mathrm{pixels}\;\mathrm{correctly}\;\mathrm{identified}\;\mathrm{as}\;\mathrm{vein}}{\mathrm{total}\;\mathrm{number}\;\mathrm{of}\;\mathrm{pixels}\;\mathrm{identified}\;\mathrm{as}\;\mathrm{vein}}\cdot 100\%$$

Accuracy is the overall correctness of the detection algorithm and it is calculated as:

(6) $$\mathrm{Accuracy}=\frac{\mathrm{sum}\;\mathrm{of}\;\mathrm{correctly}\;\mathrm{identified}\;\mathrm{pixels}}{\mathrm{total}\;\mathrm{number}\;\mathrm{of}\;\mathrm{pixels}}\cdot 100\%$$

Lastly, the total error rate is given by:

(7) $$\mathrm{Error}\;\mathrm{rate}=\frac{\mathrm{sum}\;\mathrm{of}\;\mathrm{incorrectly}\;\mathrm{identified}\;\mathrm{pixels}}{\mathrm{total}\;\mathrm{number}\;\mathrm{of}\;\mathrm{pixels}}\cdot 100\%$$

Performance evaluation results are gathered in Table 1.

On the other hand, after evaluating the data registered from the TOF camera with respect to the collected ground truth, we obtained that the position errors measured for the defined control points go from 2.1 to 3.5 mm in the *x*-axis, from 1.8 to 4.6 mm in the *y*-axis and from 0.1 to 9.9 mm in the *z*-axis, with a mean error of 2.5 mm in the *x*-axis, 2.9 mm in the *y*-axis and 3.1 mm in the *z*-axis. Note that the *x*-axis coincides roughly with the transverse axis of the hand or forehand, the *y*-axis with the longitudinal axis, and the *z*-axis with the optical axis of the TOF camera. Table 2 summarizes these results.

Lastly, the response time of the proposed algorithm was been evaluated on a 2.60 GHz Intel^®^ Core™ i5-4210M CPU with 8 GB of RAM memory. The average time for vein extraction using the adapted maximum curvature method was 1.2 s and 588 ms for the method based on the k-means clustering, whereas the average time for the registration procedure was 140 ms. That is, the total time for automatic detection and localization of peripheral subcutaneous veins with the proposed system is between 728 ms and 1.34 s.

## 4. Discussion

Performance evaluation results show that detection method based on the adapted maximum curvature exhibits a slightly better performance in terms of accuracy and total error rate than the method based on the k-means. This is due to the intrinsic nature of the algorithm, which provides less pixels identified as vein, but with a very high level of correctness. On the other hand, the method based on the k-means presents a much higher true positive rate detection, while keeping the accuracy and the total error rate quite close to the values provided by the adapted maximum curvature method. That means that a higher number of pixels are correctly classified as vein, and although incorrect detections also increase, they do not demerit the algorithm accuracy. With a higher number of correctly identified pixels, it is easier to determine some characteristics of veins, such as their width, which can help in selecting the best target vein for the automatic insertion of catheters. In addition, as during the mapping that takes place in the registration procedure some true positive detection points can be lost, it is better to have a high positive detection rate that guarantees proper registration of the pixels identified as vein.

Therefore, both methods attain a high level of correctness in identifying the image pixels that belong to the peripheral subcutaneous veins, but the method based on the k-means clustering fits better as input for the subsequent registration procedure, improving the overall performance result. It is also important to remark that ground truth labelling of images that were used for the performance evaluation was done manually, and this process is not 100% free from mistakes. Consequently, labelling errors can also contribute to shorten the performance values.

Regarding performance results related to the TOF data registration procedure, it is relevant to note that the TOF camera is characterized by suffering from flying pixels and noise. In addition, the registration algorithm is dealing with a correspondence between images of 144 × 176 pixels from the TOF camera and images of 636 × 508 pixels from the SWIR camera. Moreover, manual measurement of distances for ground truth data is not exempt from errors, which could explain the appearance of some isolated maximum errors, far from the mean values. Thus, the mean position errors obtained during the experimental test are quite acceptable, but should be further improved in the near future for increasing the reliability of the stated application.

On the other hand, the response time of the proposed algorithm satisfactorily fulfils the requirements for real-time applications. This coupled with its affordable cost and its reduced size and compactness makes it suitable for clinical routine use. Therefore, performance evaluation results highlight the feasibility and the potential benefits of the proposed solution.

## 5. Conclusions

This paper proposed a multisensory approach for the detection and localization of peripheral subcutaneous veins as a first step for achieving a guidance system that can be used in the future for automatic catheter insertion with the help of a robotic system. The solution includes a SWIR camera for acquiring reflectance measurements in the NIR region that are used for detecting the image pixels that belongs to the subcutaneous veins, a TOF camera that provides fast acquisition of Cartesian coordinates for enabling the localization of the target veins and a NIR lighting source for improving the contrast between the subcutaneous veins and the surrounding tissues.

The algorithm designed for the proposed multisensory system includes the vein features extraction from the SWIR image and the registration of the detection results with the data provided by the TOF camera, in such a way that range data can be associated to the image pixels identified as veins. For vein features extraction, two methods were proposed and evaluated experimentally, one based on the adaptation of the maximum curvature method and another based on the *k*-means clustering, both combined with several preprocessing steps for improving detection performance. Although both methods exhibit satisfactory detection accuracy, the method based on the k-means clustering fits better for the posterior registration process, given its higher TP rate.

Preliminary experimental results demonstrate the feasibility of the proposed design and highlight the potential benefits of the solution. However, future work should be directed to enhance the localization performance in order to achieve a more reliable application.

## Figures and Tables

**Table 1:** Detection performance evaluation.

Test		TP	Accuracy	Error Rate
1	Method based on adapted max. curvature	21.9%	97.0%	3.0%
	Method based on k-means	86.7%	96.5%	3.5%
2	Method based on adapted max. curvature	16.0%	97.3%	2.7%
	Method based on k-means	72.3%	97.0%	3.0%
3	Method based on adapted max. curvature	22.0%	97.1%	2.9%
	Method based on k-means	80.9%	96.1%	3.9%

**Table 2:** Position errors from the 3D registered data.

Axis	Minimum Error	Maximum Error	Mean Absolute Error
*x*	2.1 mm	3.5 mm	2.5 mm
*y*	1.8 mm	4.6 mm	2.9 mm
*z*	0.1 mm	9.9 mm	3.1 mm
